# Anatomy and evaluation of the optic nerve head

**DOI:** 10.5935/0004-2749.20220080

**Published:** 2025-02-11

**Authors:** Lilian F Machado, Rafael L. Furlanetto, Carolina P. B. Gracitelli

**Affiliations:** 1 Department of Ophthalmology and Vision Science, Glaucoma Service, Escola Paulista de Medicina, Universidade Federal de São Paulo, São Paulo, SP, Brazil; 2 Centro de Estudos Alcides Hirai, Ver Mais Oftalmologia, Vinhedo, SP, Brazil

**Keywords:** Glaucoma, Optic nerve, Optic nerve disease, Optic disk, Glaucoma, Nervo óptico, Doença do nervo óptico, Disco óptico

## Abstract

Evaluation of the optic disc is important for the correct diagnosis and follow-up of optic neuropathies, especially glaucoma. The characteristics of the optic disc depend on various factors, including demographic and population aspects, and analysis of these characteristics may vary according to the methods used. The size and format of the neural rim along with the nerve fiber layer are important to the clinician’s judgment regarding the susceptibility of the subject to develop glaucoma. In this study, we reviewed the literature to summarize the main methods and its characteristics in the evaluation of the optic nerve head.

## Abstract (pt)

A avaliação do disco óptico é de suma importância para o diagnostico correto e acompanhamento de neuropatias ópticas, especialmente o glaucoma. Características do disco óptico dependem de uma grande variedade de fatores, incluindo aspectos demográficos e populacionais, e também podem variar de acordo com os métodos usados. Tamanho e formato da rima neurorretiniana junto com a camada de fibras nervosas são importantes ao julgamento clinico a respeito da susceptibilidade do paciente desenvolver glaucoma. Nesse estudo, nós revisamos a literatura para resumir os principais métodos e suas características para a avaliação da cabeça do nervo óptico.

## INTRODUCTION

Glaucoma is a multifactorial optic neuropathy whose diagnosis depends on the assessment of risk factors and particularities of the eye examination^(1)^. The knowledge of the nuances of the optic nerve head has played a main role in the early perception of the disease, in addition to the functional characterization of the visual field^(2)^.

Knowledge about the optic disc is extremely important for detecting optic neuropathies^(3)^. Evaluations of the size and shape of the optic papilla provide information that increases the diagnostic chances and helps monitor diseases such as glaucoma^(4)^. Anatomical, epidemiological, and technical variations may interfere in the evaluation of the optic disc and neuroretinal rim. The size and characteristics of the disc influence the probability of diagnosis of glaucomatous disease^(3,4)^.

As one of the most prevalent optic neuropathies and causes of blindness in the world, glaucoma became an important condition that requires prompt detection by structural analysis of the optic nerve head^(1)^. The characteristics of the papilla, such as its size and shape, and its neuroretinal rim and cup may correlate with glaucoma^(4)^. However, these variables may differ depending on the evaluation method used^(3)^, which limits comparisons between studies using different technologies. In this narrative review, we critically analyzed the anatomical aspects of the optic disc and the tools for proper evaluation.

## SIZE AND FORMAT OF THE OPTIC DISC

### Size

The disc area correlated with axial size and refractive error in some studies^(5-8)^. After the age of 10 years, age loses its influence on the anatomy of the optic disc, and a previous study showed no relationship between age and sex, height, visual acuity, and the depth of the anterior chamber^(9)^. Zangwill et al. reported that larger optic discs had a stronger correlation with African- American ethnicity than with other races^(6)^. Previous studies^(5-8)^ also reported that a larger disc has a thinner neuroretinal rim and larger cup. On the other hand, the Ocular Hypertension Treatment Study suggests that African Americans’ increased susceptibility to developing glaucoma is influenced by other variables, as this study ruled out disc size as a risk factor of the disease. With respect to the influence of sex on the optic disc features, the literature is inconclusive about the existence of a disparity between the sizes of the optic discs in men and women^(10)^.

Although a strong correlation was observed between aging and the development of glaucoma, the relationship between age and increased or decreased optic nerve dimensions has not been proven.

Regarding the refractive error, myopia is a welldocumented risk factor for development of glaucoma. In comparison with emmetropic globes, myopic eyes, mainly those with ammetropia >-5 diopters, may have tilted, elongated, and larger optic discs, probably due to the stretching during eye growth^(3,4,11)^.

### Optic disc and cup

The disc shape is slightly vertically oval and has no correlation with sex, age, body weight, or height. A study^(12)^ with an open-angle glaucoma group suggests that glaucoma susceptibility does not depend on the shape of the optic disc. Primary macrocups generally lead to macrodiscs but may also appear as a glaucoma disc. Highly myopic patients may present secondary highly myopic macrocups due to the stretching of the optic nerve head^(12)^. The physiological cup tends to have a horizontal oval shape, and the presence of a cup augmented vertically cup or enlarged in all directions are characteristics of glaucomatous neuropathy, as they may suggest loss of nerve fibers. However, not all large cups are representative of glaucoma, as a large disc may have a large cup and a healthy neuroretinal rim. Thus, classifying the size of the disc is fundamental for the interpretation of findings under the suspicion of glaucoma. The proportion between the sizes of the optic disc and its cup, so-called cup-to-disc ratio, is probably one of the most popular features noted by clinicians in the evaluation of the optic nerve head. Large cups in large optic discs tend to be more frequently diagnosed as glaucomatous nerves; however, small discs with initial or even moderate glaucoma may be inadvertently considered as normal. According to Zangwill et al., the cup-to-disc ratio is quite variable in the population^(6)^, and when the cup-to-disc ratio is adequate, the proportions of normal and glaucomatous eyes overlap. The contour of the cup must be determined during the background biomicroscopic examination to avoid relying only on color and to monitor the paths of vessels and their final kink on the cup edge.

## NEURORETINAL RIM

Individuals without optic neuropathy usually have a pink or orange neuroretinal rim of uniform diameter. The rim seems to decrease with age and with increased intraocular pressure. One of the findings of the study by Jonas et al. that included normal eyes is the wellknown pattern of neural rim in which the inferior area is larger than the superior area, followed by the nasal and, finally, the temporal area, which is called the ISNT pattern^(7)^. The configuration of the neural rim may be of greater value than the simple analysis of the optic disc area in the detection of initial glaucoma. A generalized decrease in the rim associated with increased cupping may represent an early sign of glaucomatous damage. The neuroretinal rim loss in the glaucoma disc generally initiates in the inferior or inferotemporal and superior or superotemporal sectors^(10)^. However, as the anatomy of the optic nerve head varies widely among individuals, some features may confound the analysis of the neuroretinal rim. Oblique insertion of the optic disc in nearsighted individuals distorts the expected normal distribution of the fibers and may violate the ISNT rule, which reduces the temporal rim, indicating localized thinning. In addition, the gray crescent, most usually found in the temporal or lower temporal region of the neural rim, is a normal variation that can be interpreted as pathological thinning and is more frequent in individuals of African descent^(3)^. The neuroretinal rim is an essential parameter for monitoring the disc, and the susceptible regions of the rim must be carefully evaluated^(9)^.

### Correlation of the optic disc size with the neuroretinal rim

No consensus has been reached regarding the correlation between the size of the disc and the amount of nerve fibers. However, Jonas et al. suggested that the larger the optic disc, the greater the number of layers of nerve fibers^(7)^. The large clinical trial Blue Mountains Eye Study showed that the cup-to-disc ratio is positively associated with the diameter of the disc; thus, larger optic discs also have larger vertical cup-to-disc ratios^(13)^. The disc size correction required for quantifying the optic disc dimensions must be addressed when analyzing these variables for the detection of glaucomatous damage^(14)^. Whether disc size is an independent risk factor of glaucoma is controversial^(4)^. The disc size is known to vary widely between populations, individuals, and the eyes of the same individual^(15)^. An alternative to evaluation method for the neuroretinal rim is the disc damage likelihood scale (DDLS), a grading system for estimating the degree of optic nerve glaucomatous damage^(16)^. The scale divides discs into sizes and is based on the width of the rim. A Brazilian study demonstrated good accuracy in the evaluation of the vertical and horizontal cup/disc in the discrimination of healthy and glaucomatous eyes using the DDLS^(16)^. The susceptibility to glaucomatous neuroretinal rim loss may be partially caused by the distance to the exit of the central retinal vessel trunk on the lamina cribrosa surface. The glaucomatous loss of the neuroretinal rim can be related to the longer distance to the central retinal vessel trunk exit^(3)^.

## PALLOR OF THE OPTIC DISC

The area of the cupping and pallor of the optic disc are usually considered the same in most normal eyes; however, this cannot be true in eyes with glaucoma or neurological disease. According to Schwartz^(17)^, the area of pallor is larger or equal to the area of the cup in eyes with neurological diseases, and the opposite occurs in glaucomatous eyes. In several glaucoma discs, cupping extends to the area of color contrast. During the ophthalmologic examination, the deviation of small vessels indicates cupping, whereas the absence of these vessels indicates pallor^(17)^.

## LAMINA CRIBOSA

Some of the main signs of cribriform lamina are represented by posterior protrusion, presence of striations, and pore exposition. Although they are more commonly found in glaucomatous eyes, they are not specific and can also be found in normal optic nerves. The presence of these signs especially with other glaucoma signs such as cup asymmetry must raise a suspicion of a glaucomatous optic nerve^(18,19)^.

## VASCULAR SIGNS

The variability of the blood vessel position in the optic disc does not allow a specific standardization. However, some aspects such as a vascular trunk exit dislocated nasally may be indicative of glaucoma. The distance between the retinal vascular trunk and the neuroretinal rim may be one of the factors that affect the susceptibility to local rim loss. Vascularization of the optic disc depicts some less-specific signs of glaucoma but may help in the early detection of the disease. Some of these signs are as follows^(3)^: 1) Baring of the circumlinear blood vessels, which suggests rim loss in the sector. As the cupping increases, the circumlinear vessel no longer rests on the rim edge and becomes bared. 2) Bayonet vessel: a sign usually observed in more advanced stages of glaucoma in which the path of the vessel draws a 90° angle when entering the edge of the optic nerve as a result of substantial localized neural loss. 3) Overpass vessel: vessel without support due to loss of neural rim. 4) Disc hemorrhage: rare in normal eyes, with only 4% to 7% of glaucoma patients having intradisc or peridisc hemorrhage over the course of the disease^(20)^, which typically progressively disappears within a few weeks. Although this feature is not pathognomonic of the disease, it represents a major risk factor for developing glaucoma in hypertensive ocular patients and for the progression of glaucoma. Disc hemorrhage is strongly suggestive of glaucoma diagnosis when associated with increased intraocular pressure; however, it is also a common feature in normotensive patients with glaucoma^(3)^.

## PARAPILLARY CHORIORETINAL ATROPHY

**Figure 1:**
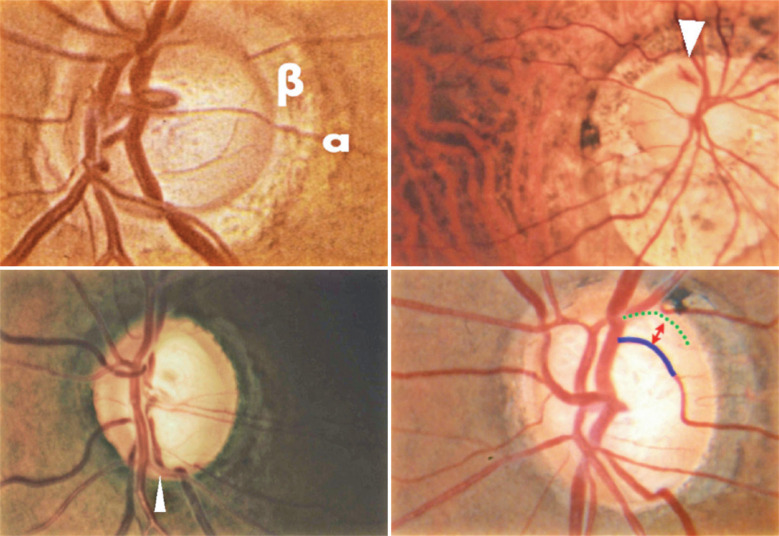
Peripapillary atrophy: alpha and beta zones. Disc hemorrhage in a patient with highly myopic glaucoma (arrow). Bottom: Bayonet vessel (arrow). Baring of the circumlinear vessels.

Peripapillary atrophy represents a complete loss of the retinal pigment epithelium (RPE) and is one of the disc characteristics that may be helpful in differentiating between glaucomatous and non-glaucomatous optic nerves and is associated with axial myopia^(21)^. Peripheral peripapillary chorioretinal atrophy (alpha zone), an irregular hypopigmentation and hyperpigmentation of the RPE, and intimated thinning of the choriorretinal tissue layer, are present in almost all normal eyes^(22)^. The beta zone, an inner atrophy of the RPE and choriocapillaris but with intact Bruch’s membrane (BM), is more often found in glaucomatous eyes^(23)^. The gamma zone is parapapillary atrophy without BM and between the optic disc border and the edge of the BM that may be more associated to scleral stretching in myopia^(24)^. Figure 1 Illustrates peripapillary atrophy alpha and beta zones with disc hemorrhage in a patient with highly myopic glaucoma. The bottom image shows the bayonet vessel. The baring of the circumlinear vessels is also shown.

## OPTIC DISC EVALUATION METHOD

The size of the optic disc image depends on the magnification of the instrument and the properties of the eye itself. Refractive error, corneal curvature, and axial length can affect measurements^(4)^. Some indirect measurement methods are indirect biomicroscopy with distinctive accessory lenses (78, 90, or 20 diopters), ophthalmoscopy with confocal scanning laser, and optical coherence tomography (OCT). The only direct method of estimating optic nerve size would be during vitreoretinal surgery. All methods have advantages, limitations, and magnification corrections, which will be described in the following sections and in table 1.

**Table 1:** Comparison between the methods

	Advantages	Disadvantages
**Slit-lamp indirect biomicroscopy**	- Stereoscopic vision	- Difficult to perform in children and non-cooperative patients
	- Magnifies the nerve	- May need mydriasis
**Stereoscopic photography**	- Most reliable documenting method	- Interobserver variability
	- Gold standard for assessing progression	- High image quality required
**optic coherence tomography**	- Fast, noninvasive, and noncontact-does not require a reference plan	- Artifacts: speckle noise, segmentation and alignment errors, low signal quality, and media opacities
	- High sensitivity and specificity (90%)	-good functional/structural correlation
**Ultrasonography**	- Noninvasive method - Accessible - Useful in opaque media	- Few optic nerve details

### Slit-lamp indirect biomicroscopy

The procedure is performed dynamically with a slit lamp by using ancillary lenses. The light beam is projected coinciding with the edges of the disc vertically and horizontally, taking care to exclude the scleral ring. The quantitative measurement can be obtained considering the correction of each lens (90, 78, and 60 diopters). The slit-lamp measurement is multiplied by 1.3, 1.2, and 0.88, respectively^(3)^. This method magnifies the nerve and provides good illumination and stereoscopic vision, but can be difficult to perform in children and non-cooperative patients. In such cases, indirect ophthalmoscopy can be used, but important details of the nerves may be missed. In 2019, Colicchio et al. demonstrated better diagnostic accuracy for glaucoma and visualization of the cup and disc when examining the optic nerve with background biomicroscopy under mydriasis, suggesting that this examination be performed with a dilatation of the pupil whenever possible^(25)^.

### Stereoscopic photography

This is the most reliable method for documenting glaucomatous optic atrophy. Comparison of serialcolored stereoscopic photographs of an optic disc is considered the gold standard for assessing the progression of glaucoma^(26-28)^. Surprisingly, studies have shown that glaucoma specialists do not routinely obtain stereoscopic photographs of discs in clinical practice^(29,30)^. Two photos are taken in sequence with an Allen separator or simultaneously with two cameras in slightly different angles. The advantages of this method are as follows: 1) not as expensive as other methods; 2) colors help identify the limits of the cup and neural rim and detect hemorrhages and peripapillary atrophies; and 3) allows comparison of the photos over time^(13,31)^. The main disadvantages are the considerable interobserver variability and high image quality required^(31)^. A recent study by Mwanza et al. assessed the agreement between indirect biomicroscopy and stereoscopic photographs taken by glaucoma specialists. In this study, 505 optic disc photos were analyzed, and a wide variation between measurements was found, which suggested poor agreement among almost all the parameters examined. Following this rationale, the authors proposed that glaucoma should be monitored with a single method instead of migrating from one method to another^(31-36)^.

### Optical coherence tomography

**Figure 2:**
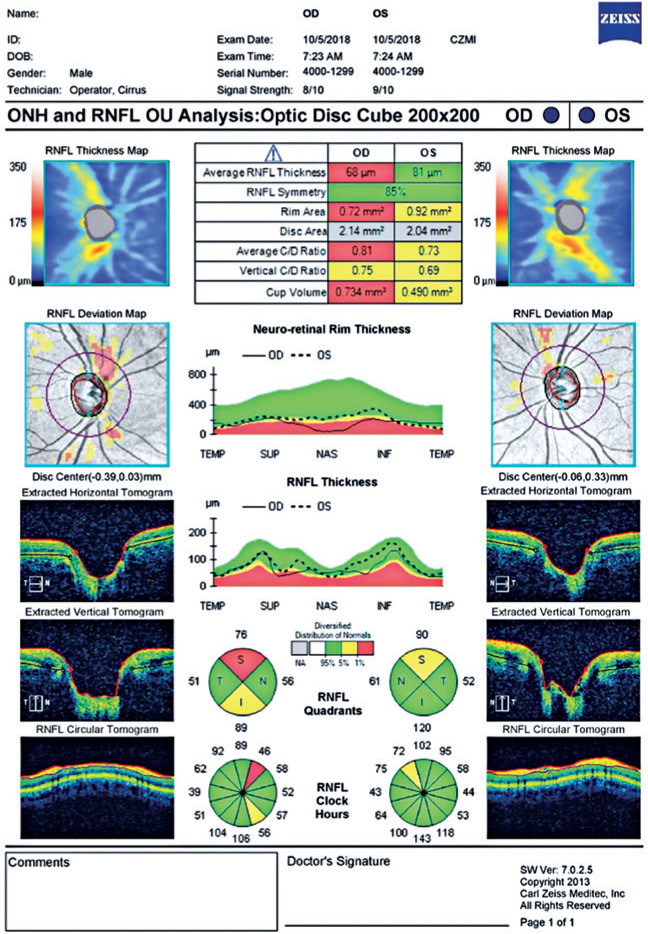
Cirrus 500 printout of the optical coherence tomography image.

A low-coherence infrared light beam generates crosssectional images of the optic nerve head and forms a sequence of signals similar to that generated by a B-mode ultrasonography device. It is a noninvasive, noncontact technology and does not require a reference plane^(37)^. Although it allows visualization of the microstructures in the optic nerve head and provides a different analysis of the optic disc, the most used and studied parameter in glaucoma is still the peripapillary retinal nerve fiber layer thickness. In this analysis, the thickness measurements of the nerve fiber layer around the optic disc in each image were compared with those in the normative database. The advent of spectral-domain OCT and then Swept-Source OCT led to the enhancement of image resolution, increase in the signal-to-noise ratio, reduction in acquisition time, wide-angle scans with large posterior pole areas, and improvement in measurement reproducibility. Despite these improvements, OCT is prone to limitations such as artifacts as speckle noise, segmentation and alignment errors, low signal quality, and media opacities^(38)^. Ocular diseases such as myopia, age-related macular degeneration, or macular druse may induce other artifacts and lead to difficult interpretations. Studies have reported that measuring topographic optic disc parameters and neurofiber thickness may be less effective in the diagnosis of glaucomatous or non-glaucomatous eyes with high myopia^(37)^. Segmentation of the optic nerve head has improved with a new software reference of the fovea’s position and BM as the anatomical parameter. The BM opening minimal rim width has a close association with functional changes and better ability of detecting early glaucoma^(39)^. OCT has a sensitivity and specificity of approximately 90%^(31)^ and good functional/structural correlation, depending on the number of patients included and the parameters studied. Recently, an application of OCT has been extended to angiography and blood flow measurement. OCT angiography (OCT-A) is used to evaluate retinal vascular abnormalities and has been found to reduce retinal perfusion on the optic nerve head and peripapillary retina in glaucomatous patients^(40)^. The main limitations of OCT-A are due to the artifacts from blood flow fluctuations. Although OCT-A represents a new technology, its clinic value to glaucoma evaluation remains to be determined. The consensus of the World Glaucoma Association considers OCT as the best imaging device for nerve fiber layer measurement to detect and monitor the optic nerve damage in primary open-angle glaucoma. Figure 2 depicts an OCT Cirrus 500 printout.

### Ultrasonography

**Figure 3:**
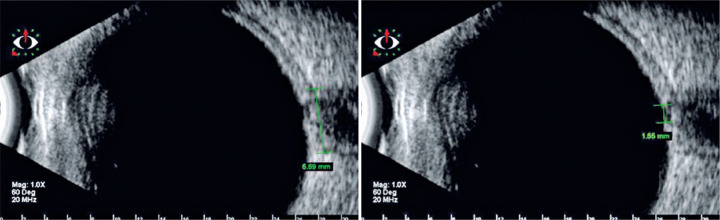
Ultrasonography image of the optic nerve head.

Ultrasonography has been widely used in investigations related to the diameter and, contiguously, sheath of the retrobulbar optic nerve in glaucomatous optic neuropathy^(41-43)^. Reduced sectional dimensions in patients with glaucoma as compared with healthy indivi duals were attributed to the loss of nerve fibers that occurs in this disease. It is a noninvasive and easily accessible method, which can be used to investigate the disease, especially in cases where the morphology of the optic disc does not allow a conclusive diagnosis or in the presence of opaque media, which makes visualization of the optic nerve head difficult or even impossible^(42,43)^. Figure 3 illustrates an example of an optic nerve head observed on ultrasonography.

Glaucoma is an ocular disease that ophthalmologists must be knowledgeable of. The optic disc anatomy and evaluation are key to obtaining useful information from examinations. The aim of this study was to summarize the current literature on the analysis methods used for characterizing optic disc nerve head. Anatomy studies reported little information about the evaluation of the glaucomatous optic disc signs and epidemiology but showed the great variability and evolution of the evaluation methods. Some authors first used slit-lamp biomicroscopic examination and even ultrasonography to analyze and establish a suspicion of glaucoma in the optic nerve. Although stereophotos remain important for the documentation and follow-up of the optic nerve head, other examinations such as the more recent OCT provide more advanced additional quantitative and qualitative information about a suspected glaucomatous optic nerve head. In conclusion, the combination of careful anatomical examination and updated analysis methods may provide the best glaucoma evaluation of the optic disc.

## References

[1] Weinreb RN, Aung T, Medeiros FA. (2014). The pathophysiology and treatment of glaucoma: a
review. JAMA.

[2] O’Connor DJ, Zeyen T, Caprioli J. (1993). Comparisons of methods to detect glaucomatous optic nerve
damage. Ophthalmology.

[3] Jonas JB, Budde WM, Panda-Jonas S. (1999). Ophthalmoscopic evaluation of the optic nerve
head. Surv Ophthalmol.

[4] Hoffmann EM, Zangwill LM, Crowston JG, Weinreb RN. (2007). Optic disk size and glaucoma. Surv Ophthalmol.

[5] Oliveira C, Harizman N, Girkin CA, Xie A, Tello C, Liebmann JM (2007). Axial length and optic disc size in normal eyes. Br J Ophthalmol.

[6] Zangwill LM, Weinreb RN, Berry CC, Smith AR, Dirkes KA, Coleman AL, Confocal Scanning Laser Ophthalmoscopy Ancillary Study to the Ocular
Hypertension Treatment Study (2004). Racial differences in optic disc topography: baseline results
from the confocal scanning laser ophthalmoscopy ancillary study to the
ocular hypertension treatment study. Arch Ophthalmol.

[7] Jonas JB, Gusek GC, Naumann GO. (1988). Optic disc, cup and neuroretinal rim size, configuration and
correlations in normal eyes. Invest Ophthalmol Vis Sci.

[8] Nangia V, Matin A, Bhojwani K, Kulkarni M, Yadav M, Jonas JB. (2008). Optic disc size in a population-based study in central India: the
Central India Eye and Medical Study (CIEMS). Acta Ophthalmol.

[9] Gandhi M, Dubey S. (2013). Evaluation of the optic nerve head in glaucoma. J Curr Glaucoma Pract.

[10] Jonas JB, Budde WM, Lang P. (1998). Neuroretinal rim width ratios in morphological glaucoma
diagnosis. Br J Ophthalmol.

[11] Seider MI, Lee RY, Wang D, Pekmezci M, Porco TC, Lin SC. (2009). Optic disk size variability between African, Asian, white,
Hispanic, and Filipino Americans using Heidelberg retinal
tomography. J Glaucoma.

[12] Jonas JB, Gründler AE, Papastathopoulos KI. (1998). Optic disc dimensions, body length, and body
weight. Br J Ophthalmol.

[13] Healey PR, Mitchell P, Smith W, Wang JJ. (1997). Relationship between cup-disc ratio and optic disc diameter: the
Blue Mountains Eye Study. Aust N Z J Ophthalmol.

[14] Jonas JB, Bergua A, Schmitz-Valckenberg P, Papastathopoulos KI, Budde WM. (2000). Ranking of optic disc variables for detection of glaucomatous
optic nerve damage. Invest Ophthalmol Vis Sci.

[15] Mansour AM. (1991). Racial variation of optic disc size. Ophthalmic Res.

[16] Kara-José AC, Melo Jr, LA (2017). Esporcatte BL, Endo AT, Leite MT, Tavares IM. The disc damage
likelihood scale: diagnostic accuracy and correlations with cup-to-disc
ratio, structural tests and standard automated perimetry. PLoS One.

[17] Schwartz B. (1973). Cupping and pallor of the optic disc. Arch Ophthalmol.

[18] Nicolela MT, Drance SM. (1996). Various glaucomatous optic nerve appearances: clinical
correlations. Ophthalmology.

[19] Broadway DC, Nicolela MT, Drance SM. (1999). Optic disk appearances in primary open-angle
glaucoma. Surv Ophthalmol.

[20] Airaksinen PJ, Mustonen E, Alanko HI. (1981). Optic disc hemorrhages. Analysis of stereophotographs and
clinical data of 112 patients. Arch Ophthalmol.

[21] Jonas JB, Schiro D, Naumann GO. (1993). [The retinal nerve fiber layer in normal and glaucoma
eyes]. Ophthalmologe.

[22] Jonas JB, Naumann GO. (1989). Parapapillary chorioretinal atrophy in normal and glaucoma eyes.
II. Correlations. Invest Ophthalmol Vis Sci.

[23] Manalastas PI, Belghith A, Weinreb RN, Jonas JB, Suh MH, Yarmohammadi A (2018). Automated beta zone parapapillary area measurement to
differentiate between healthy and glaucoma eyes. Am J Ophthalmol.

[24] Jonas JB, Fernández MC, Naumann GO. (1992). Glaucomatous parapapillary atrophy. Occurrence and
correlations. Arch Ophthalmol.

[25] Colicchio D, Terenzi LA, Rocha JA, Sousa AK, Almeida Jr, ED, Moreno PA (2020). Comparison of fundus biomicroscopy examination of the optic nerve
head with and without mydriasis. Ophthalmic Res.

[26] Kass MA, Heuer DK, Higginbotham EJ, Johnson CA, Keltner JL, Miller JP (2002). The Ocular Hypertension Treatment Study: a randomized trial
determines that topical ocular hypotensive medication delays or prevents the
onset of primary open-angle glaucoma. Arch Ophthalmol.

[27] Leske MC, Heijl A, Hyman L, Bengtsson B. (1999). Early Manifest Glaucoma Trial: design and baseline
data. Ophthalmology.

[28] Medeiros FA, Alencar LM, Zangwill LM, Bowd C, Sample PA, Weinreb RN. (2009). Prediction of functional loss in glaucoma from progressive optic
disc damage. Arch Ophthalmol.

[29] Fremont AM, Lee PP, Mangione CM, Kapur K, Adams JL, Wickstrom SL (2003). Patterns of care for open-angle glaucoma in managed
care. Arch Ophthalmol.

[30] Hertzog LH, Albrecht KG, LaBree L, Lee PP. (1996). Glaucoma care and conformance with preferred practice patterns.
Examination of the private, community-based ophthalmologist. Ophthalmology.

[31] Mwanza JC, Grover DS, Budenz DL, Herndon LW, Nolan W, Whiteside-de Vos J (2017). A comparison of cup-to-disc ratio estimates by fundus
biomicroscopy and stereoscopic optic disc photography in the Tema Eye
Survey. Eye (Lond).

[32] Strouthidis NG, Garway-Heath DF. (2008). New developments in Heidelberg retina tomograph for
glaucoma. Curr Opin Ophthalmol.

[33] Wollstein G, Garway-Heath DF, Hitchings RA. (1998). Identification of early glaucoma cases with the scanning laser
ophthalmoscope. Ophthalmology.

[34] Janknecht P, Funk J. (1994). Optic nerve head analyser and Heidelberg retina tomograph:
accuracy and reproducibility of topographic measurements in a model eye and
in volunteers. Br J Ophthalmol.

[35] Rao HB, Sekhar GC, Babu GJ, Parikh RS. (2009). Clinical measurement and categorization of optic disc in glaucoma
patients. Indian J Ophthalmol.

[36] Fallon M, Valero O, Pazos M, Antón A. (2017). Diagnostic accuracy of imaging devices in glaucoma: A
meta-analysis. Surv Ophthalmol.

[37] Gracitelli CP, Abe RY, Medeiros FA. (2015). Spectral-Domain Optical Coherence Tomography for Glaucoma
Diagnosis. Open Ophthalmol J.

[38] Dong ZM, Wollstein G, Schuman JS. (2016). Clinical Utility of Optical Coherence Tomography in
Glaucoma. Invest Ophthalmol Vis Sci.

[39] Chauhan BC, O’Leary N, AlMobarak FA, Reis AS, Yang H, Sharpe GP (2013). Enhanced detection of open-angle glaucoma with an anatomically
accurate optical coherence tomography-derived neuroretinal rim
parameter. Ophthalmology.

[40] Liu L, Jia Y, Takusagawa HL, Pechauer AD, Edmunds B, Lombardi L (2015). Optical Coherence Tomography Angiography of the Peripapillary
Retina in Glaucoma. JAMA Ophthalmol.

[41] Jonas JB, Schmidt AM, Müller-Bergh JA, Naumann GO. (1995). Optic nerve fiber count and diameter of the retrobulbar optic
nerve in normal and glaucomatous eyes. Graefes Arch Clin Exp Ophthalmol.

[42] Dichtl A, Jonas JB. (1996). Echographic measurement of optic nerve thickness correlated with
neuroretinal rim area and visual field defect in glaucoma. Am J Ophthalmol.

[43] Beatty S, Good PA, McLaughlin J, O’Neill EC. (1998). Echographic measurements of the retrobulbar optic nerve in normal
and glaucomatous eyes. Br J Ophthalmol.

